# The Influence of Polymers on the Supersaturation Potential of Poor and Good Glass Formers

**DOI:** 10.3390/pharmaceutics10040164

**Published:** 2018-09-21

**Authors:** Lasse I. Blaabjerg, Holger Grohganz, Eleanor Lindenberg, Korbinian Löbmann, Anette Müllertz, Thomas Rades

**Affiliations:** 1 Department of Pharmacy, University of Copenhagen, Universitetsparken 2, 2100 Copenhagen, Denmark; lasse.blaabjerg@sund.ku.dk (L.I.B.); korbinian.loebmann@sund.ku.dk (K.L.); anette.mullertz@sund.ku.dk (A.M.); thomas.rades@sund.ku.dk (T.R.); 2 Idorsia Pharmaceuticals Ltd., Hegenheimermwattweg 91, CH-4123 Allschwil, Switzerland; Eleanor.lindenberg@idorsia.com; 3 Faculty of Science and Engineering, Åbo Akademi University, Tykistökatu 6A, 20521 Turku, Finland

**Keywords:** glass forming ability, amorphous, degree of supersaturation, precipitation inhibitor, pKa

## Abstract

The increasing number of poorly water-soluble drug candidates in pharmaceutical development is a major challenge. Enabling techniques such as amorphization of the crystalline drug can result in supersaturation with respect to the thermodynamically most stable form of the drug, thereby possibly increasing its bioavailability after oral administration. The ease with which such crystalline drugs can be amorphized is known as their glass forming ability (GFA) and is commonly described by the critical cooling rate. In this study, the supersaturation potential, i.e., the maximum apparent degree of supersaturation, of poor and good glass formers is investigated in the absence or presence of either hypromellose acetate succinate L-grade (HPMCAS-L) or vinylpyrrolidine-vinyl acetate copolymer (PVPVA64) in fasted state simulated intestinal fluid (FaSSIF). The GFA of cinnarizine, itraconazole, ketoconazole, naproxen, phenytoin, and probenecid was determined by melt quenching the crystalline drugs to determine their respective critical cooling rate. The inherent supersaturation potential of the drugs in FaSSIF was determined by a solvent shift method where the respective drugs were dissolved in dimethyl sulfoxide and then added to FaSSIF. This study showed that the poor glass formers naproxen, phenytoin, and probenecid could not supersaturate on their own, however for some drug:polymer combinations of naproxen and phenytoin, supersaturation of the drug was enabled by the polymer. In contrast, all of the good glass formers—cinnarizine, itraconazole, and ketoconazole—could supersaturate on their own. Furthermore, the maximum achievable concentration of the good glass formers was unaffected by the presence of a polymer.

## 1. Introduction

The increased focus on combinatorial chemistry and high-throughput screening in drug discovery has led to an increase in poorly aqueous soluble drug candidates, with low and variable bioavailability in pharmaceutical development [1]. In an effort to overcome this challenge, various enabling formulation techniques are currently being investigated. Among these, amorphization of the drug has been shown to increase the apparent solubility of drugs, i.e., leading to supersaturation of the drug with respect to the most stable crystalline form, thereby increasing the driving force for absorption of the drug [2,3]. The ease with which such drugs can be amorphized is known as their glass forming ability (GFA).

A classification system of GFA based on the recrystallization tendency of drugs during cooling and heating cycles has previously been proposed [4]. Drugs which recrystallized during cooling of the melt at a cooling rate of 20 K/min belonged to GFA class 1, whereas GFA class 2 drugs did not recrystallize during cooling of the melt at 20 K/min, but recrystallized in the following heat cycle at a heating rate of 10 K/min. Finally, GFA class 3 drugs neither recrystallized during cooling of the melt at 20 K/min nor during the following heat cycle at 10 K/min [4]. Classes 1, 2 and 3 represent poor, modest and good glass formers, respectively. This classification system based on arbitrary categories was further substantiated in a later study that revealed that the drugs can be classified according to their inherent crystallization tendency independent of the predefined categories [5]. The new classification system is based on the critical cooling rate, i.e., the slowest cooling rate during melt quenching that results in amorphization of the drug [5]. GFA class 1 drugs inherently have critical cooling rates above 750 K/min, GFA class 2 drugs have critical cooling rates between 10 and 20 K/min and GFA class 3 drugs have critical cooling rates below 2 K/min [5,6]. 

The dissolution of an amorphous drug may result in a “spring effect”, leading to a fast generation of a supersaturated drug concentration, with respect to the thermodynamic equilibrium solubility. However, this may be followed by rapid precipitation, which limits the potential solubility advantage of the amorphous drug [7]. Drug precipitation is a result of nucleation and crystal growth as the drug is in a thermodynamically unfavorable state when supersaturated. For a given drug, a higher degree of supersaturation increases the risk of precipitation [8,9]. 

The degree of supersaturation is defined as the ratio between the activity of the dissolved drug in a supersaturated solution and the activity of the drug in a saturated solution of the thermodynamically stable form [10]. However, the thermodynamic activity of a drug in a supersaturated solution in a fasted state simulated intestinal fluid (FaSSIF), as used in this study, is difficult to determine since the drug may become incorporated into micelles or other colloidal species present in FaSSIF [11]. Therefore, the concentration of the solubilized drug is used instead of the activity to calculate an apparent degree of supersaturation (aDS):

aDS = C_supersaturation_/C_equilibrium_ (1)

where C_supersaturation_ is the concentration of the drug in a supersaturated solution and C_equilibrium_ is the concentration of the drug in a saturated solution.

Polymers have been exploited in drug development to stabilize the amorphous form of the drug, either by reducing the rate of nucleation and/or the rate of crystal growth of the drug in the solid state [12]. However, in contrast, it has been shown that a polymer can also promote nucleation of the drug as was shown for acetaminophen in the presence of poly(acrylic) acid compared to pure acetaminophen when investigated by hot-stage microscopy [12]. Additionally, polymers can also inhibit precipitation of the drug in a supersaturated solution, which may result in a “parachute effect”, maintaining the supersaturated concentration of the drug for a longer period of time [7,13,14]. The inhibitory effect of the polymer may simply be explained by mediation of an increase in drug solubility, which decreases the degree of supersaturation and thereby the driving force for precipitation. Furthermore, polymers can also increase the viscosity of the solution, thereby increasing steric hindrance (decreased nucleation) and decrease the effective diffusion coefficient (decreased crystal growth) of the drug [3]. Finally, polymers can interact specifically with drug molecules in solution, inhibiting both nucleation and crystal growth [15]. As the polymers may work in a multitude of ways to stabilize the drug in supersaturation, it is difficult to predict the effect of a specific polymer [16].

Hypromellose acetate succinate (HPMCAS) and vinylpyrrolidine-vinyl acetate copolymer (PVPVA64) are amongst the most often used polymers in recently marketed amorphous drug products [17]. Several studies have investigated the influence of polymer concentration on the inhibitory effect of precipitation during supersaturation of drugs and found that concentrations as low as 0.001% (*w*/*v*) to be effective [18,19]. For the selection of the polymer concentrations to investigate, a more pragmatic approach is to assume that the achievable polymer concentrations in vivo range from 0.05% to 0.5% (*w*/*v*) based on a 1 g formulation containing 10% (*w*/*w*) polymer dispersed in the small intestinal fluid volume in the fasted state (105 ± 72 mL) [20].

To study supersaturation of a drug, the solvent shift method is widely used [21,22,23,24]. In this method, supersaturation is induced by dissolving the drug in an organic water-miscible solvent in which it is highly soluble and then transferring the solution into an aqueous acceptor medium where the drug has a low solubility. Van Eerdenbrugh et al. (2014) studied the supersaturation propensity, i.e., the time until precipitation at a fixed aDS, of 50 different drugs by the use of a solvent shift method and monitored the time until crystallization of the drug in the supersaturated solution occurred. The study proposed a classification system based on three categories: class I drugs crystallized within 150 s, class II drugs within 1 h and class III drugs did not crystallize within 1 h. A later study also applied the solvent shift method to induce supersaturation of six different drugs at a fixed concentration (100 mg/mL) in phosphate buffer and proposed another classification system using four categories: “Fast drugs” crystallized within 15 min, “moderate-fast” drugs within 3 h, “moderate-slow” drugs within 6 h and “slow” drugs between 6 and 12 h [22]. References [21,22] were unsuccessful in correlating their respective classification systems of supersaturation propensity of a drug with its GFA, which may be explained by the use of a fixed aDS to study the supersaturation propensity.

A recent study proposed a standardized supersaturation and precipitation method (SSPM) to determine the inherent supersaturation potential of a drug, i.e., the maximum achievable aDS [23]. In this solvent shift method, a highly concentrated solution of the drug is titrated into an acceptor medium in which the drug is much less soluble. As the titration induces supersaturation the drug in the acceptor medium, the drug will eventually precipitate and the maximum achievable aDS, i.e., the potential of the drug to supersaturate, is determined as the concentration at which precipitation occurs during titration. Three lower concentrations are additionally tested relative to the maximum achievable aDS and the times to precipitation are determined [23]. It has been shown that the influence of a low amount of organic solvent (e.g., 2.0% *v*/*v* dimethyl sulfoxide used in the SSPM) on the solubility of the drug can be neglected [25].

A later study showed that GFA of a drug and its supersaturation potential, i.e., the maximum achievable aDS, may indeed be correlated [24]. Here, the inherent GFA of a drug was classified according to the previously mentioned classification system based on the critical cooling rate of the drug and the inherent supersaturation potential of the same drug was determined by the SSPM previously developed [5,23]. The study showed that the supersaturation potential of GFA class 3 drugs was higher than that of GFA class 2 drugs [24]. As mentioned above, a few studies have attempted to correlate the GFA of a drug with its potential to supersaturate, but to the authors’ knowledge, no studies have previously investigated this potential correlation in the presence of polymers.

This study aims to further investigate the inherent supersaturation potential of poor and good glass formers in the absence and presence of a polymer. The inherent GFA of the selected drugs is determined by melt quenching using a method previously proposed [5]. The supersaturation potential of the respective drugs is determined by the use of the SSPM proposed by Palmelund et al. (2016).

## 2. Materials and Methods

### 2.1. Materials

Itraconazole, naproxen and phenytoin were obtained from Chemie Brunschwig (Basel, Switzerland). Cinnarizine, ketoconazole, probenecid, sodium chloride, sodium hydroxide and sodium phosphate dihydrate were obtained from Sigma-Aldrich (Steinheim, Germany). Dimethyl sulfoxide was obtained from VWR Chemicals (Søborg, Denmark). SIF powder was obtained from Biorelevant (South Croydon, UK). HPMCAS-L was gifted by Dow Wolf Cellulosics (Bomlitz, Germany) and PVPVA64 was obtained from BASF (Basel, Switzerland). All materials were used as received.

### 2.2. Determination of Thermal Stability

The thermal stability of the drugs was determined using a TGA Discovery (TA Instrument, New Castle, DE, USA) as reported previously [5]. Data analysis was performed using Trios software version 3.3.0.4055 (TA Instruments-Waters LLC, New Castle, DE, USA) to calculate the weight loss between 298 K and the drug’s melting point (T_m_) + 20 K. The experiments were conducted in triplicate.

### 2.3. Determination of Glass Forming Ability by Melt Quenching

The critical cooling rate was determined using a DSC 8500 (PerkinElmer, Zürich, Switzerland) as reported previously [5]. The critical cooling rate, i.e., the slowest cooling rate that resulted in a full amorphization of the sample was determined using the selected cooling rates shown in Appendix A. Data analysis was performed using Pyris software version 11.0.0.0449 (PerkinElmer, Zürich, Switzerland). The experiments were conducted in triplicate.

### 2.4. Determination of pKa

The pKa of the drugs was determined using a SiriusT3 apparatus (Sirius Analytical Instruments Ltd., Forest Row, UK) fitted with an Ag/AgCl double-junction reference electrode and an overhead stirrer at a temperature of 298 K controlled by the Sirius software. UV-metric pKa titrations were carried out in 1.5 mL ion-strength-adjusted water (0.15 M KCl), titrating with either 0.5 M HCl or 0.5 M KOH using nitrogen as purge gas. Titrations were carried out in the pH range of pH 2 to pH 12 with an initial concentration of the drug of 30 mM. All measurements were conducted in triplicate.

### 2.5. Preparation of Fasted State Simulated Intestinal Fluid

FaSSIF was prepared from a commercially available SIF powder as specified by the manufacturer (Biorelevant, South Croydon, UK). Phosphate buffer was prepared with 3.954 mg/mL monobasic sodium phosphate, 0.420 mg/mL NaOH, and 6.186 mg/mL NaCl dissolved in purified water (SG Ultra Clear UV 2002, Evoqua water Technologies LLC, Barsbüttel, Germany) and adjusted to pH 6.5 using either 1 M NaOH or 1 M HCl. SIF powder was dissolved in the phosphate buffer (2.24 mg/mL) and stirred for 2 h at ambient temperature before use.

### 2.6. Determination of Equilibrium Solubility

The equilibrium solubility at 310 K of the investigated compounds was determined by the use of a µ-DISS Profiler (Pion, Billerica, MA, USA) using the experimental settings shown in Appendix B. An excess amount of the crystalline drug was added to the vessels containing either FaSSIF (pH 6.5), 0.05 or 0.5% (*w*/*v*) PVPVA64 in FaSSIF or 0.05 or 0.5% (*w*/*v*) HPMCAS-L in FaSSIF. The pH of the solution was measured by a Sension+ PH31 electrode from Hach (Düsseldorf, Germany). The equilibrium solubility was taken as the maximum drug concentration, no later than 24 h into the experiment. The solution was filtered using a 0.25 µm cellulose acetate Q-MAX^®^ RR syringe filter (Frisenette Aps, Knebel, Denmark) to remove all particles in the media, which may interfere with the in-situ UV measurements due to light scattering. The experiments were conducted using at least three replicates for each drug.

The equilibrium solubility (after 24 h) in pH adjusted FaSSIF was determined using reverse phase high-pressure liquid chromatography (HPLC). 200 μL saturated drug solution in a 0.5 mL Pro cup was centrifuged at 23,000 g (298 K) for 20 min in an Eppendorf Centrifuge 5417R (Eppendorf, Basel, Switzerland). 10 μL of the sample was diluted with 990 μL acetonitrile and analysed by HPLC using a SPD-M30A detector, SIL-30AC auto-sampler, LC-30AD pump and CTO-20A oven from Shimadzu (Reinach, Switzerland). The mobile phase consisted of acetonitrile and 0.05% trifluoroacetic acid and the flow rate was 1.5 mL/min. The injection volume was 1.0 μL (except for itraconazole, where the injection volume was 20 μL) and the separation was conducted using a Phenomenex Kinetex 2.6 μm EVO C18 column (100 Å, 50 × 2.1 mm) (Brechbühler, Schlieren, Switzerland). The column oven was set to 322 K and standard curves were prepared in the range of 0.06 μg/mL to 1000 μg/mL for each drug (*r*^2^ > 0.99). The investigated UV wavelength for each drug was: cinnarizine (252 nm), itraconazole (258 nm), ketoconazole (225 nm), naproxen (272 nm), phenytoin (218 nm) and probenecid (250 nm). The experiments were conducted in triplicate.

### 2.7. Determination of the Supersaturation Potential

Supersaturation of the drugs in FaSSIF with and without polymers (0.05 or 0.5% (*w*/*v*) HPMCAS-L or PVPVA64) was induced using a solvent shift method developed previously [23]. The concentration of the drug in the stock solution was found by determining the maximum concentration in the acceptor media before precipitation at a total DMSO concentration of 2% (*v*/*v*). Standard curves for all drugs were prepared by adding aliquots (20–50 µL) of the drug dissolved in dimethyl sulfoxide (1–50 mg/mL) into FaSSIF until immediate precipitation was observed. Precipitation was detected by a shift in the baseline of the UV spectrum, diversion from linearity between absorbance and concentration of the drug and by visual inspection.

The stock solution was used to induce supersaturation in FaSSIF. 200 µL of stock solution was spiked into 10 mL FaSSIF (310 K), and stirred with a cross bar magnet at 100 ± 3 rpm to ensure complete mixing within the 10 s. The concentration of the drug in solution and the time until precipitation were measured in-situ using the second derivative of the UV-absorbance in the µ-DISS Profiler until precipitation occurred (or for a maximum of 24 h). The second derivate of the UV-absorbance was used to minimize interference from particle light scattering. The induction time (t_ind_) was taken as the time at which the concentration of the supersaturated solution had decreased by 2.5%. The experiment was conducted for each drug using a minimum of three replicates.

### 2.8. Statistical Analysis

The equilibrium solubility, supersaturation potential and maximal concentration during supersaturation are given as the mean ± SD. The samples were compared by analysis of variance (ANOVA) using Microsoft Excel 2013 (Microsoft, Redmond, WA, USA). A statistical *p* value > 0.05 was considered significant. Data in figures denoted with an asterisk (*) are statistically significantly different from the control.

## 3. Results

### 3.1. Determination of GFA of Drugs

The GFA of a drug can be classified experimentally by determination of the critical cooling rate using melt quenching in a differential scanning calorimeter or minimal milling time using vibrational ball milling [5,6]. For classification using melt quenching, the thermal stability upon melting of the six selected drugs was initially investigated by thermogravimetric analysis. All drugs degraded by less than 5% (*w*/*w*) upon melting (data not shown) and were therefore deemed suitable for classification by melt quenching. In this study naproxen, phenytoin and probenecid could not be made fully amorphous even when using cooling rates of the melt of 750 K/min, which means these three drugs have critical cooling rates higher than 750 K/min and therefore belong to class 1 with respect to GFA, i.e., these drugs are poor glass formers. In contrast, cinnarizine, itraconazole and ketoconazole could be prepared in an amorphous form using cooling rates of the melt below 2 K/min and are therefore class 3 drugs with respect to GFA, i.e., good glass formers (Table 1). These findings are in line with previous classifications reported in the literature [5,6]. It should be noted that even though it was not possible to fully amorphize GFA class 1 drugs on their own, several studies have shown that GFA class 1 drugs, such as naproxen, can form glass solutions using a polymer, another drug or an amino acid as co-former [26,27,28].

### 3.2. Determination of Equilibrium Solubility with and without Polymer

**Figure 1:**
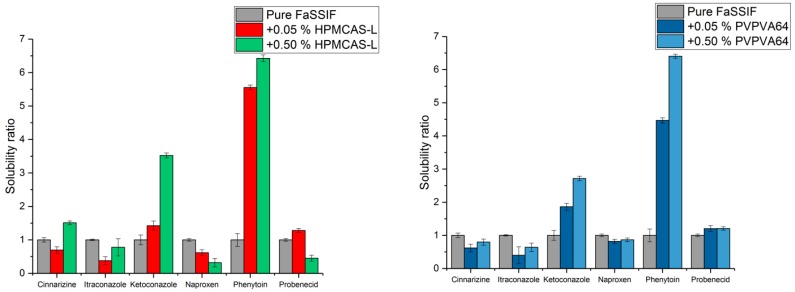
Solubility ratio of the model drugs in fasted state simulated intestinal fluid (FaSSIF) with hypromellose acetate succinate L-grade (HPMCAS-L) (**left**) and vinylpyrrolidine-vinyl acetate copolymer (PVPVA64) (**right**) (drug in pure fasted state simulated intestinal fluid (FaSSIF) is given a ratio of 1).

The equilibrium solubility of the drugs was determined to calculate their respective apparent degree of supersaturation. Solubility ratios of the investigated drugs in presence of either HPMCAS-L or PVPVA64 relative to their solubility in pure FaSSIF are shown in Figure 1. The respective equilibrium solubility of the drugs in FaSSIF is shown in Table 2 and the equilibrium solubility of the drugs in the respective media is shown in Appendix C.

For cinnarizine, itraconazole, ketoconazole and phenytoin, the solubility of the drug was higher in presence of 0.5% (*w*/*v*) polymer compared to 0.05% (*w*/*v*) polymer for both PVPVA64 and HPMCAS-L (Figure 1). In contrast, for naproxen and probenecid, the solubility was similar in presence of either 0.5 or 0.05% (*w*/*v*) PVPVA64. Furthermore, the solubility of the two drugs was lower in presence of 0.5% (*w*/*v*) HPMCAS-L compared to 0.05% (*w*/*v*) HPMCAS-L (Figure 1).

It is well known that the addition of a polymer can increase the solubility of a drug as seen for phenytoin and ketoconazole in presence of either 0.05 or 0.5% (*w*/*v*) HPMCAS-L or PVPVA64 compared to pure FaSSIF [15]. Unexpectedly, for probenecid and cinnarizine, the solubility only increased in presence of 0.05 and 0.5% (*w*/*v*) HPMCAS-L, but not PVPVA64, compared to pure FaSSIF. Furthermore, for the remaining drug:polymer combinations of probenecid and cinnarizine, as well as any combination of polymer with naproxen and itraconazole, the solubility was either similar or decreased with respect to their solubility in pure FaSSIF (Figure 1).

FaSSIF prepared as described by the manufacturer has a low buffer capacity and for an ionisable drug, the solubility may be affected by changes to the pH of the medium. Determination of the pH of FaSSIF showed that the addition of 0.05 and 0.5% (*w*/*v*) HPMCAS-L decreased the pH of FaSSIF from 6.5 to 6.3 and 5.8, respectively. In contrast, the addition of PVPVA64 (0.05 or 0.5% (*w*/*v*)), as expected, did not affect the pH. Furthermore, due to the poor buffer capacity of FaSSIF, dissolution of the drugs themselves can also change pH of the medium, e.g., solvation of the acidic drug naproxen in FaSSIF decreased the pH from 6.5 to 6.0.

To separate the effect of the polymer from the effect of pH on the solubility of the drug, the solubility in pH adjusted FaSSIF (i.e., FaSSIF adjusted to the resulting pH of FaSSIF in presence of polymer) was also investigated. For cinnarizine and ketoconazole, an increase in their respective solubility was seen in presence of polymer compared to pH adjusted FaSSIF (*p* < 0.01). This implies that the addition of polymer increases the solubility of the respective drugs (Table 2). In contrast, for itraconazole, naproxen, phenytoin and probenecid, the solubility with and without polymer was similar, implying that the addition of polymer did not increase the solubility of the drugs (*p* > 0.05) (Table 2).

### 3.3. Determination of the Supersaturation Potential of Poor Glass Formers

Naproxen, phenytoin and probenecid are GFA class 1 drugs, i.e., poor glass formers. Their inherent supersaturation potential in the absence and presence of predissolved HPMCAS-L or PVPVA64 (0.05 or 0.5% (*w*/*v*)) in FaSSIF was investigated using the SSPM [23].

**Figure 2:**
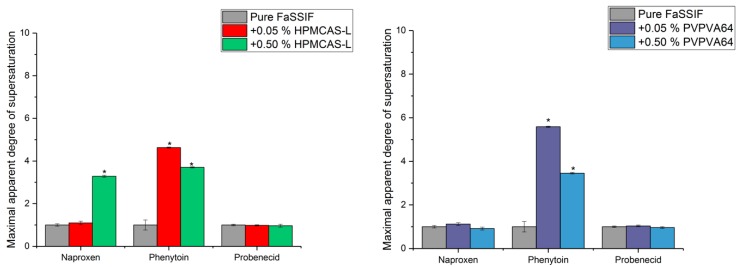
Maximum apparent degree of supersaturation of naproxen, phenytoin and probenecid in fasted state simulated intestinal fluid (FaSSIF) and in FaSSIF in the presence of hypromellose acetate succinate L-grade (HPMCAS-L) (**left**) or vinylpyrrolidine-vinyl acetate copolymer (PVPVA64) (**right**) using the solvent shift method standardized supersaturation and precipitation method (SSPM) [23].

The maximum achievable concentration from solvent shift of naproxen, phenytoin and probenecid was similar to the equilibrium solubility of the respective drugs, i.e., aDS = 1 (*p* > 0.05) (Figure 2, grey bars). This means that none of the GFA class 1 drugs could supersaturate on their own, which may be explained by the drugs either very rapidly crystallizing from a supersaturated solution or never supersaturating at all.

For naproxen in presence of 0.5% (*w*/*v*) HPMCAS-L, as well as phenytoin in presence of either 0.05 or 0.5% (*w*/*v*) HPMCAS-L a maximum aDS > 3 could be obtained using the solvent shift method (Figure 2). In contrast, for naproxen, no supersaturation was seen in the presence of 0.05% (*w*/*v*) HPMCAS-L (*p* > 0.05), and for probenecid no supersaturation was seen in the presence of either 0.05 or 0.5% (*w*/*v*) HPMCAS-L (*p* > 0.05). In the presence of PVPVA64 (0.05 or 0.5% (*w*/*v*)), a maximum aDS > 3 was obtained using the solvent shift method for phenytoin (Figure 2), whereas no supersaturation was seen for naproxen and probenecid.

In an earlier study, it was hypothesized that drugs unable to supersaturate on their own would also not be able to supersaturate in presence of excipients [30]. In contrast, the results presented in this study show that some polymers, i.e., HPMCAS-L or PVPVA64, can enable supersaturation of some of the GFA class 1 drugs and consequently, it appears that GFA class 1 drugs can supersaturate, but would on their own very rapidly recrystallize from a supersaturated solution. A similar result was found in a previous study in which the supersaturation propensity of phenytoin with and without polymer using a solvent shift method was investigated [31]. They found that phenytoin on its own would not supersaturate, whereas supersaturation of the drug was enabled in the presence of either HPMC or PVP (1.0% (*w*/*v*)). For the GFA class 1 drugs that were unable to supersaturate in the presence of HPMCAS-L or PVPVA64, it is speculated that a higher concentration of the polymer is required to enable supersaturation of the drugs, as naproxen could supersaturate in the presence of a high concentration (0.5% (*w*/*v*)) of HPMCAS-L.

**Figure 3:**
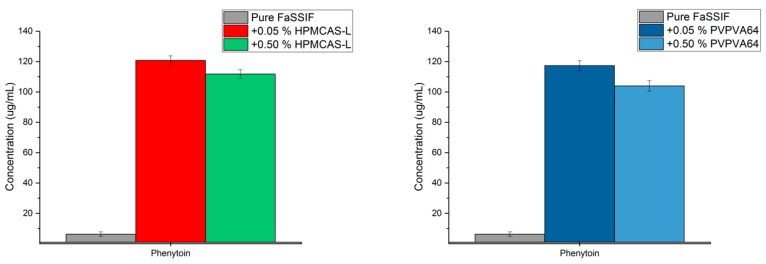
Maximum achievable concentration of phenytoin in fasted state simulated intestinal fluid (FaSSIF) and in FaSSIF in the presence of hypromellose acetate succinate L-grade (HPMCAS-L) (**left**) or vinylpyrrolidine-vinyl acetate copolymer (PVPVA64) (**right**) using the solvent shift method SSPM [23].

The solubility of a drug in FaSSIF may be increased by the presence of a polymer, which in turn would result in a decrease of the aDS. For phenytoin, the maximum aDS in the presence of 0.5% (*w*/*v*) HPMCAS-L and PVPVA64 was reduced compared to the maximum aDS in presence of the lower concentration of polymer, i.e., 0.05% (*w*/*v*) HPMCAS-L and PVPVA64. Therefore, for discussion purposes, the y-axis in Figure 3 is changed to concentration. It should be emphasized that this is simply a different way of presenting the data shown in Figure 2.

For phenytoin, it is seen that the maximum achievable concentration does not increase in the presence of increasing polymer concentration for either HPMCAS-L or PVPVA64 (Figure 3). However, the solubility of phenytoin in FaSSIF in the presence of 0.05% (*w*/*v*) HMPCAS-L increases from 26.1 µg/mL to 30.2 µg/mL in the presence of 0.5% (*w*/*v*) HPMCAS-L (Appendix C). A similar result is seen for phenytoin in the presence of PVPVA64, as the solubility in FaSSIF in the presence of 0.05% (*w*/*v*) PVPVA64 increases from 21.0 µg/mL to 30.1 µg/mL in the presence of 0.5% (*w*/*v*) PVPVA64 (Appendix C). These results confirm that the reduced supersaturation potential of phenytoin in the presence of a higher polymer concentration compared to a lower polymer concentration, i.e., 0.5% (*w*/*v*) polymer compared to 0.05% (*w*/*v*) polymer, is a result of increased solubility of the drug in presence of a polymer.

### 3.4. Determination of the Supersaturation Potential of Good Glass Formers

**Figure 4:**
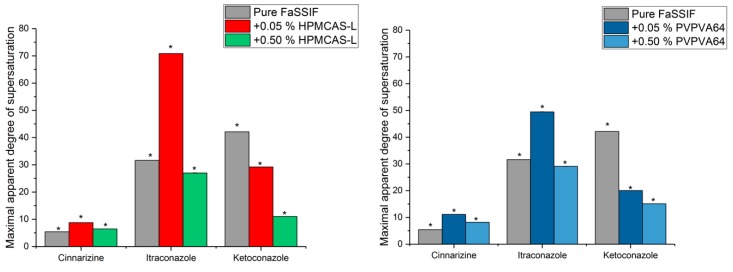
Maximum apparent degree of supersaturation of cinnarizine, ketoconazole and itraconazole in fasted state simulated intestinal fluid (FaSSIF) and in presence of hypromellose acetate succinate L-grade (HPMCAS-L) (**left**) and vinylpyrrolidine-vinyl acetate copolymer (PVPVA64) (**right**) using the solvent shift method SSPM [23]. Error bars are not visible in figure due to scaling.

Cinnarizine, itraconazole and ketoconazole are GFA class 3 drugs, i.e., good glass formers. Their supersaturation potential in the absence and presence of predissolved HPMCAS-L or PVPVA64 (0.05 or 0.5% (*w*/*v*)) in FaSSIF was investigated using the SSPM as described above [23]. A previous study showed that the three drugs could supersaturate on their own, and their respective maximum aDS were 5.4, 31.6 and 42.1, indicating the good glass formers can supersaturate on their own (Figure 4, grey bars) [24].

**Figure 5:**
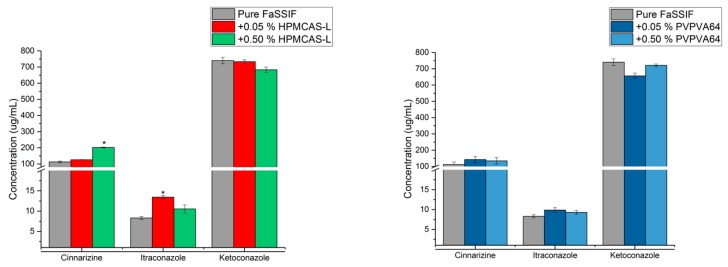
Maximum achievable concentration of cinnarizine, itraconazole and ketoconazole in fasted state simulated intestinal fluid (FaSSIF) and in FaSSIF in the presence of hypromellose acetate succinate L-grade (HPMCAS-L) (**left**) or vinylpyrrolidine-vinyl acetate copolymer (PVPVA64) (**right**) using the solvent shift method SSPM [23].

For cinnarizine and itraconazole, it was seen that the maximum aDS increased from 5.4 to 8.8 and from 31.6 to 70.8, respectively, in the presence of 0.05% (*w*/*v*) HPMCAS-L compared to pure FaSSIF (Figure 4). In contrast, the presence of a higher concentration of HPMCAS-L (0.5% (*w*/*v*)) decreased the maximum aDS of cinnarizine and itraconazole to 6.5 and 27.0, respectively compared to a lower concentration of HPMCAS-L (0.05% (*w*/*v*)). For ketoconazole, the maximum aDS in pure FaSSIF decreased from 42.1 to 29.2 and 11.0 in the presence of 0.05 and 0.5% (*w*/*v*) HPMCAS-L, respectively. The same trends were apparent for cinnarizine, itraconazole and ketoconazole in the presence of PVPVA64 compared to pure FaSSIF. Consequently, it appears that the maximum aDS of the GFA class 3 drugs can be both increased or decreased as well as be unaffected by the presence of a polymer, which is intuitively unexpected. Therefore, for discussion purposes, the y-axis in Figure 5 is changed to concentration. Once again, it is emphasized that this is simply a different way of presenting the data shown in Figure 4.

**Figure 6:**
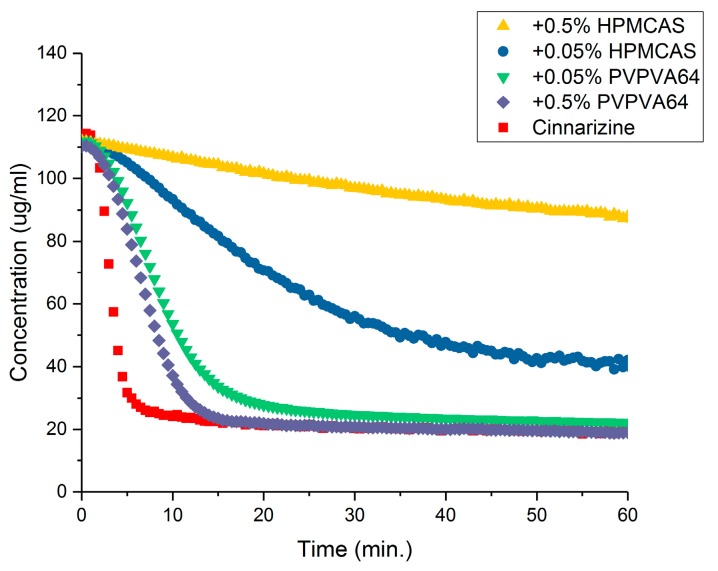
Time-concentration profiles cinnarizine in fasted state simulated intestinal fluid (FaSSIF) in the absence or presence of either hypromellose acetate succinate L-grade (HPMCAS-L) or vinylpyrrolidine-vinyl acetate copolymer (PVPVA64) using the solvent shift method SSPM [23].

As can be seen in Figure 5, the maximum achievable concentration of cinnarizine, itraconazole and ketoconazole in FaSSIF in the presence of HPMCAS-L and PVPVA64 (0.05 or 0.5% (*w*/*v*)) is not affected to a practically relevant degree. However, for e.g., cinnarizine in presence of 0.5% (*w*/*v*) HPMCAS-L, the maximum achievable concentration is higher compared to pure FaSSIF. This means that the maximum achievable concentration of a GFA class 3 drug may not be improved by addition of a polymer. This may be explained by a fast continuous crystal growth mechanism at the high degree of supersaturation of the GFA class 3 drugs, which result in the polymers being less effective compared to supersaturation at a lower degree of supersaturation [32]. However, the polymer may inhibit precipitation of the drug from a supersaturated solution, e.g., via molecular interactions between the drug and polymer or by increasing the viscosity of the solution, meaning the time that the drug can remain supersaturated may increase in the presence of a polymer as can be seen for cinnarizine in the presence of HPMCAS-L or PVPVA64 (Figure 6) [3,15,23]. From Figure 6 it is also seen that the induction time for precipitation of the drug from the supersaturated solution is very short. This means that the experimentally determined supersaturation potential using the solvent shift method SSPM is very close to the thermodynamic supersaturation potential of the drug.

### 3.5. Correlation between GFA and Supersaturation Potential of Drugs

A previous study has shown that GFA class 3 drugs have a higher supersaturation potential compared to GFA class 2 drugs, and thus there may be a correlation between GFA of a drug and its supersaturation potential [24]. In the present study, the GFA class 1 drugs naproxen, phenytoin and probenecid could not supersaturate on their own. However, for some drug:polymer combinations of naproxen and phenytoin, supersaturation of the drug was enabled by the polymer. These results indicate that GFA class 1 drugs can supersaturate, however, on their own recrystallization from the supersaturated solution occurs very rapidly. Rapid crystallization is also seen during melt quenching of the drugs, as it was not possible to prepare any of the GFA class 1 drugs in a fully amorphous form using cooling rates up to 750 K/min [5,6]. In contrast, all of the GFA class 3 drugs, cinnarizine, itraconazole and ketoconazole, could supersaturate on their own and are also easily prepared in an amorphous form, as their critical cooling rate is below 2 K/min [5,6]. Furthermore, the maximum achievable concentration of the GFA class 3 drugs was unaffected by the presence of a polymer. These findings further substantiate the correlation between GFA of a drug and its supersaturation potential.

## 4. Conclusions

In this study, the inherent GFA of six drugs was determined via melt quenching. Additionally, the inherent supersaturation potential of the same drugs was determined by the use of a solvent shift method using the μ-Diss Profiler to induce supersaturation of the drug in the absence or presence of predissolved polymer in FaSSIF. The GFA of the drugs was successfully classified according to a previously developed classification system with naproxen, phenytoin and probenecid belonging to GFA class 1 (poor glass formers) and cinnarizine, itraconazole and ketoconazole belonging to GFA class 3 (good glass formers). The GFA class 1 drugs naproxen, phenytoin, and probenecid showed no supersaturation potential on their own. However, for some combinations of naproxen and phenytoin with HPMCAS-L or PVPVA64, supersaturation of the drug was enabled by the presence of a polymer. These findings indicate that poor glass formers can supersaturate. However, on their own recrystallization from the supersaturated solution occurs very rapidly. In contrast, all of the good glass formers—cinnarizine, itraconazole and ketoconazole—can supersaturate on their own. However, the maximum achievable concentration of the GFA class 3 drugs was unaffected by the presence of the tested polymers.

## Figures and Tables

**Table 1:** T_m_, T_g_, classification of glass forming ability, critical cooling rate and pKa of the investigated drugs.

Drug	T_m_ (K) ^a^	T_g_ (K) ^a^	GFA Class	Critical Cooling Rate (K/min)	pKa (Acidic/Basic)
Naproxen	429	278 ^b^	1	>750	4.2/-
Phenytoin	569	-	1	>750	8.0/-
Probenecid	468	-	1	>750	3.3/-
Cinnarizine	393	288	3	1	-/2.0 ^c^, 7.6
Itraconazole	439	332	3	1	-/4.0
Ketoconazole	419	319	3	1	-/3.3, 6.2

^a^ Data from Blaabjerg et al. (2018); ^b^ Partial recrystallization during cooling of the melt; ^c^ Data from reference [29]. Abbreviations: Melting point (T_m_), glass transition temperature (T_g_), glass forming ability (GFA).

**Table 2:** Solubility of the investigated drugs in fasted state simulated intestinal fluid (FaSSIF), pH adjusted FaSSIF and FaSSIF + 0.5% *w*/*v* hypromellose acetate succinate L-grade (HPMCAS-L).

Drug	Solubility in FaSSIF (mM)	Solubility in pH Adjusted FaSSIF (mM)	Solubility in FaSSIF + 0.5% (*w*/*v*) HPMCAS-L (mM)
Naproxen	10.5 ± 0.4	6.1 ± 0.3 (pH 6)	6.5 ± 0.4 (pH 6)
Phenytoin	0.02 ± 0.004	0.13 ± 0.002 (pH 6)	0.12 ± 0.012 (pH 6)
Probenecid	7.1 ± 0.3	8.0 ± 0.2 (pH 6)	8.6 ± 0.3 (pH 6)
Cinnarizine	0.06 ± 0.004	0.02 ± 0.001 (pH 7)	0.08 ± 0.003 (pH 7)
Ketoconazole	0.03 ± 0.005	0.084 ± 0.006 (pH 6)	0.117 ± 0.007 (pH 6)
Itraconazole	0.001 ± 0.0001	0.001 ± 0.0002 (pH 6)	0.0006 ± 0.0001 (pH 6)

## Appendix A

**Table A1:** Applied cooling rates during melt quenching to determine GFA class.

Compound	Applied Cooling Rates (K/min)
Naproxen	750, 20
Phenytoin	750, 20
Probenecid	750, 20
Cinnarizine	750, 20, 5, 1, 0.5
Itraconazole	750, 20, 5, 1, 0.5
Ketoconazole	750, 20, 5, 1, 0.5

## Appendix B

**Table A2:** Probe tip lengths used in supersaturation studies.

Drug	Probe Tip Length (mm)
Naproxen	1
+0.05% HPMCAS-L	1
+0.50% HPMCAS-L	1
+0.05% PVPVA64	1
+0.50% PVPVA64	1
Phenytoin	1
+0.05% HPMCAS-L	1
+0.50% HPMCAS-L	1
+0.05% PVPVA64	1
+0.50% PVPVA64	1
Probenecid	1
+0.05% HPMCAS-L	1
+0.50% HPMCAS-L	1
+0.05% PVPVA64	1
+0.50% PVPVA64	1
Cinnarizine	1
+0.05% HPMCAS-L	1
+0.50% HPMCAS-L	1
+0.05% PVPVA64	1
+0.50% PVPVA64	1
Itraconazole	5
+0.05% HPMCAS-L	5
+0.50% HPMCAS-L	5
+0.05% PVPVA64	5
+0.50% PVPVA64	5
Ketoconazole	2
+0.05% HPMCAS-L	2
+0.50% HPMCAS-L	2
+0.05% PVPVA64	2
+0.50% PVPVA64	2

## Appendix C

**Table A3:** Solubility of drugs in fasted state simulated intestinal fluid (FaSSIF) in absence and presence of either hypromellose acetate succinate L-grade (HPMCAS-L) or vinylpyrrolidine-vinyl acetate copolymer (PVPVA64) together with final pH values of the respective media.

Medium	Naproxen (ug/mL)	Phenytoin (μg/mL)	Probenecid (μg/mL)	Cinnarizine (μg/mL)	Itraconazole (μg/mL)	Ketoconazole (μg/mL)
FaSSIF	2412.0 ± 96.5	4.7 ± 0.9	2028.3 ± 79.3	20.7 ± 1.4	0.5 ± 0.1	17.6 ± 2.6
	pH (6.0)	pH (6.4)	pH (6.7)	pH (6.4)	pH (6.4)	pH (6.5)
+0.05% HPMCAS-L	768.5 ± 14.1	26.1 ± 1.6	2603.4 ± 126.6	14.4 ± 0.1	0.2 ± 0.1	25.1 ± 2.3
	pH (5.7)	pH (6.4)	pH (5.5)	pH (6.4)	pH (6.4)	pH (6.3)
+0.50% HPMCAS-L	1488.3 ± 87.1	30.2 ± 3.0	2499.0 ± 96.1	31.3 ± 1.0	0.4 ± 0.1	62.0 ± 3.9
	pH (5.1)	pH (6.4)	pH (5.2)	pH (5.8)	pH (5.7)	pH (5.6)
+0.05% PVPVA64	1970.6 ± 81.6	21.0 ± 1.4	2440.1 ± 202.7	12.8 ± 0.5	0.2 ± 0.1	32.8 ± 2.2
	pH (6.0)	pH (6.4)	pH (6.0)	pH (6.4)	pH (6.4)	pH (6.4)
+0.50% PVPVA64	2093.2 ± 64.8	30.1 ± 1.6	2299.0 ± 92.1	16.5 ± 0.6	0.3 ± 0.	47.8 ± 2.0
	pH (5.8)	pH (6.4)	pH (5.8)	pH (6.4)	pH (6.4)	pH (6.4)
